# Feasibility of mixed nut challenges in children with suspicion of multiple tree nut allergies

**DOI:** 10.1186/2045-7022-5-S3-P85

**Published:** 2015-03-30

**Authors:** Francine C Van Erp, IL Kok, MF Van Velzen, AC Knulst, CK Van der Ent, Y Meijer

**Affiliations:** 1 Department of Paediatric Pulmonology and Allergology, Wilhelmina Children's Hospital, University Medical Centre Utrecht, Utrecht, The Netherlands; 2 Internal Medicine and Dermatology, Department of Dietetics, University Medical Centre Utrecht, The Netherlands; 3 Department of (Paediatric) Dermatology and Allergology, University Medical Centre Utrecht, Utrecht, The Netherlands

## Background

In children without any previous ingestion of nuts multiple sensitization to tree nuts is common. To minimize the risk of accidental reactions, those children are advised to exclude all nuts from their diets. Multiple food challenges would be needed to determine the presence of clinical relevant tree nut allergies, which is practically impossible. A pilot study to determine the feasibility of a mixed nut challenge in children with suspicion of multiple tree nut allergies was performed.

## Methods

Children with previous negative hazelnut challenge and a lifelong nut free diet and / or sensitization to one or more tree nuts underwent an open mixed nut challenge. The mixture contained 6 gram of four or six tree nuts and applesauce. The challenge consisted of increasing portions of the mixture and was followed by the ingestion of each whole tree nut separately and when indicated a reintroduction schedule at home. Feasibility regarding safety, reactions during challenge, tolerance of the challenge material, satisfaction of the parents, reintroduction and dietary restrictions after challenge were evaluated.

## Results

We included 18 children with a mean (SD) age of 10.2 (3.4) years. Mixed nut challenges were well accepted and conclusive in 16 (89%) children and had a negative outcome in 11 (61%) children. No life threatening reactions were observed, reactions were classified up to Sampson grade 2. Overall 12 children (67%), including one child with inconclusive outcome, could reintroduce one or more tree nuts at home. Five children (28%) could return to a diet without any nut related restrictions.

## Conclusion

Mixed nut challenges are well accepted, safe and an efficient way to exclude multiple tree nut allergies especially in children with a lifelong nut free diet and low suspicion of allergy.

